# Clinical Epidemiological Analysis of the Genotypic Spectrum and Mortality Risk in Carbapenem‐Resistant *Klebsiella pneumoniae* (CRKP) Infections

**DOI:** 10.1155/cjid/1529426

**Published:** 2026-01-06

**Authors:** Qiongfang Zhang, Ze Fang, Bo Fang, Hailing Zeng, Jiaojiao Xu

**Affiliations:** ^1^ Department of Infection Control, Zhongjiang County People’s Hospital, Deyang, Sichuan, China; ^2^ Department of Endocrinology, Zhongjiang County People’s Hospital, Deyang, Sichuan, China; ^3^ Administrative Office, Zhongjiang County People’s Hospital, Deyang, Sichuan, China; ^4^ Department of Laboratory Medicine, Zhongjiang County People’s Hospital, Deyang, Sichuan, China

**Keywords:** carbapenem-resistant *Klebsiella pneumoniae*, CRKP, epidemiology, genotype, KPC, meta-analysis, mortality, OXA-48

## Abstract

**Background:**

Carbapenem‐resistant *Klebsiella pneumoniae* (CRKP) has become a global public health threat, with significantly increased morbidity and mortality among high‐risk inpatients. High‐risk genotypes of CRKP, primarily including *K. pneumoniae* carbapenemase (KPC), oxacillinase‐48–like (OXA‐48–like), New Delhi metallo‐β‐lactamase (NDM), imipenemase (IMP), and Verona integron–encoded metallo‐β‐lactamase (VIM) types (hereafter referred to as KPC, OXA‐48–like, NDM, IMP, and VIM, respectively) as defined by international consensus due to their association with enhanced transmission, drug resistance, and mortality risk, have emerged as key challenges in clinical practice. However, the impact of different genotypic types of CRKP on mortality risk and their regional epidemiology remain unclear.

**Methods:**

This systematic review and meta‐analysis were conducted according to the PRISMA guidelines and registered in PROSPERO (CRD420251150672). PubMed, Embase, and Web of Science were systematically searched for original studies up to September 9, 2025. Eligible studies reported genotypic types of CRKP (such as KPC, OXA‐48–like, NDM, IMP, and VIM) and patient mortality. The pooled odds ratio (OR) and 95% confidence interval (CI) were calculated using a random‐effects model. Subgroup analyses were performed by the genotype and region. Risk of bias was assessed using the ROBINS‐I tool.

**Results:**

A total of 58 studies involving various countries and regions were included. The pooled analysis showed that CRKP infection with high‐risk genotypes—defined in this study primarily as KPC and OXA‐48–like types due to their strong association with increased transmission, drug resistance, and mortality—was significantly associated with increased all‐cause mortality (pooled OR = 3.08, 95% CI: 2.44–3.88, *p* < 0.0001; *I* ^2^ = 63.4%). Subgroup analysis revealed that KPC‐type CRKP infection had a higher mortality risk (pooled OR = 3.57, 95% CI: 2.82–4.53), with the effect more pronounced in China (OR = 3.85) than in other countries (OR = 3.04). The OXA‐48–like genotype also showed increased mortality risk (OR = 2.57, 95% CI: 1.47–4.50), while evidence for other genotypes such as NDM, IMP, and VIM was limited. Sensitivity analysis and publication bias tests supported the robustness of the results. GRADE assessment indicated moderate evidence for KPC but lower quality for other genotypes.

**Conclusions:**

High‐risk genotypes of CRKP—defined in this study as KPC and OXA‐48–like due to their strong association with increased transmission, drug resistance, and mortality—are associated with significantly increased mortality risk in infected patients, especially in China. These findings support risk stratification and personalized management of high‐risk patients and provide evidence for regional prevention and control strategies. Further multicenter and mechanistic studies are warranted to improve clinical management and patient outcomes.

## 1. Introduction

### 1.1. Research Background and Current Status

In recent years, the global trend of infections caused by multidrug‐resistant bacteria has become increasingly severe, posing a major public health threat worldwide. Carbapenem‐resistant bacteria, especially carbapenem‐resistant *Klebsiella pneumoniae* (CRKP), have experienced frequent outbreaks in many parts of the world, bringing great challenges to hospital infection control and clinical treatment [1, 2]. Data show that the detection rate of CRKP isolates continues to rise in some high‐incidence countries (such as China and India), particularly among high‐risk populations including intensive care units (ICUs), neonatology, and organ transplantation patients [3–5]. CRKP has become one of the most representative highly drug‐resistant Gram‐negative bacteria worldwide. Its multidrug‐resistant and pandrug‐resistant [PDR] characteristics (i.e., extensively drug‐resistant [XDR], PDR) significantly limit the options for available antibiotics, leading to treatment failure and increased mortality [1–4, 6].

*Klebsiella* is one of the most common pathogens causing hospital‐acquired infections. Due to its strong drug resistance and high pathogenicity, CRKP has gradually replaced traditional pathogens as the “difficult bacteria” representative in hospital infections [1, 2]. The proportion of CRKP in nosocomial infection events such as bloodstream infections, pneumonia, and urinary tract infections has been increasing year by year. Its isolation rate and resistance rate are particularly prominent in high‐risk departments or populations such as ICUs, transplant patients, and neonates [3, 5, 7, 8]. For example, in China, the recent epidemic of CRKP is mainly dominated by ST11 and KPC‐2 types, which are widely distributed and often lead to explosive nosocomial transmission, endangering the lives of critically ill patients [4, 9].

High‐risk genotypes of CRKP, as defined in this study, refer to molecular types with clear epidemiological evidence for increased transmission, drug resistance, and mortality risk—primarily KPC and OXA‐48–like types according to international consensus. Other genotypes such as NDM, IMP, and VIM are also included in the analysis but are less frequently reported worldwide.

### 1.2. Clinical Significance and Challenges of CRKP

CRKP infections are mostly seen in hospitalized patients, especially those in ICUs, neonatology, organ transplant recipients, and high‐risk populations with multiple underlying diseases (such as liver cirrhosis, tumors, and diabetes) [1, 4, 5, 7]. The ICU environment, due to the critical condition of patients, frequent invasive procedures, and extensive use of antibiotics, is a “disaster area” for the prevalence and outbreaks of CRKP. According to large‐scale multicenter epidemiological investigations, the isolation rate of CRKP in ICUs is significantly higher than in general wards [4, 7, 9]. Neonates and preterm infants, as well as kidney and solid organ transplant recipients, are also high‐risk groups for CRKP infection, and these infections often lead to serious complications and nosocomial outbreaks [3, 5, 8].

Because of its extremely strong drug resistance, CRKP infection is generally considered to be closely associated with a higher risk of mortality, which is particularly prominent in critically ill patients, those with underlying diseases, and organ transplant recipients [3, 7, 8]. Current studies indicate that the risk of death in patients infected with CRKP is much higher than in those infected with carbapenem‐sensitive strains, and most deaths occur in clinical situations where outbreaks happen and treatment options are limited [1, 5]. Due to the limited availability of antibiotics, the treatment of CRKP infections often faces multiple challenges, including a broad spectrum of resistance, heavy dependence on combination therapy, and some strains even being resistant to “last‐resort” drugs such as tigecycline and colistin [2, 4]. This not only increases the disease burden and length of hospitalization for patients but also leads to greater consumption of medical resources and greater difficulty in infection control, imposing huge pressure on both hospital and social healthcare systems [2, 5].

It is worth emphasizing that although there have been many reports on the overall mortality risk of CRKP infections, there is still a lack of evidence‐based summaries regarding the differences in mortality risk among different molecular genotypes of CRKP and their influencing factors. This is precisely the area where clinical and public health fields urgently need breakthroughs at present [1, 3].

### 1.3. Research Gaps, Significance, and Innovation

Although the epidemiology, resistance mechanisms, and clinical prognosis of CRKP infections have received widespread attention in recent years, most existing studies are single‐center or regional cohorts with limited sample size and analytical dimensions.

In this study, high‐risk genotypes are defined as *K. pneumoniae* carbapenemase (KPC) and oxacillinase‐48–like (OXA‐48–like) types, in line with current international molecular epidemiology guidelines, because of their strong association with nosocomial outbreaks, multidrug resistance, and adverse clinical outcomes. Other molecular genotypes such as New Delhi metallo‐β‐lactamase (NDM), imipenemase (IMP), and Verona integron‐encoded metallo‐β‐lactamase (VIM) are also considered, though with fewer reported cases.

Most of them focus on the association between overall resistance of CRKP and clinical outcomes, lacking systematic reviews and quantitative analyses of the relationship between different molecular resistance genotypes (such as KPC, OXA‐48–like, NDM, IMP, and VIM) and the mortality risk of infected patients [1, 3, 4]. In addition, outcome indicators are not unified in some literature, and there is a relative lack of discussion on the clinical significance of genotype differences, which brings challenges to the precise identification and risk stratification of high‐risk patients [5, 8].

This study explores the association between major resistance genotypes of CRKP (KPC, OXA‐48–like, NDM, IMP, etc.) and the mortality risk of infected patients, with a particular focus on the inclusion and analysis of relevant data from China, reflecting the regional epidemiological characteristics and practical significance for local clinical practice [4, 9]. By multidimensional analysis of the relationships among resistance genotypes, geographic distribution, and mortality risk, this study aims to provide evidence‐based support for optimizing CRKP infection management and prevention strategies in high‐incidence areas (such as China) and also to provide a theoretical basis for understanding the association between different resistance mechanisms and clinical heterogeneity [2, 3].

### 1.4. Research Objective

The aim of this study is to systematically evaluate the mortality risk of patients infected with different resistance genotypes, with a particular focus on analyzing the epidemiological characteristics and prognostic differences in China, clarifying the association between major genotypes and adverse outcomes, and providing evidence‐based support for risk stratification management of high‐risk patients and optimization of regional prevention and control strategies.

## 2. Methods

### 2.1. Study Design and Registration

This study was conducted in strict accordance with the Preferred Reporting Items for Systematic Reviews and Meta‐Analyses (PRISMA) guidelines. The study protocol was registered in the international systematic review registration platform PROSPERO (Registration Number: CRD420251150672) to ensure the transparency and reproducibility of the research.

### 2.2. Literature Search and Selection

This study systematically searched three major databases: PubMed, Embase, and Web of Science. The search was conducted up to September 9, 2025, with no language restrictions. Five researchers independently performed the initial screening and full‐text review of the literature. Relevant studies were selected based on predefined inclusion and exclusion criteria, and studies that did not meet the criteria were excluded. By reading the titles, abstracts, and full texts, the final studies included in the analysis were determined.

### 2.3. Inclusion and Exclusion Criteria

#### 2.3.1. Inclusion Criteria

1. The study population consisted of patients with confirmed CRKP infection.
2. The study clearly detected and reported the molecular genotype of CRKP (such as KPC, NDM, OXA‐48–like, VIM, and IMP), allowing for genotyping or subgroup statistics.
3. At least one all‐cause mortality indicator was provided (such as 30‐day mortality, in‐hospital mortality, or mortality during the follow‐up period) or could be calculated based on the original data.
4. Study types included clinical cohort studies (prospective or retrospective), case–control studies (CCS), or cross‐sectional studies and other original studies.
5. Data were complete, and the number of genotype‐positive cases and corresponding deaths could be obtained, or relevant data could be inferred from the original text.
6. The literature was published in English or Chinese, and the full text was available.

#### 2.3.2. Exclusion Criteria

1. Studies with subjects that were in vitro experiments, animal experiments, mechanistic studies or only conducted molecular epidemiological analysis on isolates/environmental samples without providing clinical outcomes.
2. Studies that did not detect or report specific CRKP genotypes, and only generally reported CRKP infections.
3. Studies that did not report mortality or number of deaths and from which related outcomes could not be indirectly calculated.
4. Nonoriginal studies, such as reviews, meta‐analyses, case reports, conference abstracts, and book chapters.
5. Studies with seriously incomplete data or data that could not be used for analysis or multiple publications from the same cohort/center (only the most complete information was retained).
6. Other studies that did not meet the inclusion purpose, such as studies with extremely small sample sizes (e.g., only 1 case), or only methodological papers.

### 2.4. Data Extraction

In this study, data extraction was independently performed by four researchers. The extracted information included the first author, year of publication, region, study type, sample size, CRKP molecular genotype, mean age, patient type, follow‐up duration, all‐cause deaths, and the corresponding odds ratio (OR) with 95% confidence interval (CI). In cases of missing data, the researchers contacted the authors for Supporting Information or completed the data through indirect calculation. All extracted data were reviewed by the first author to ensure their accuracy and consistency.

### 2.5. Quality Assessment

The Risk of Bias in Non‐randomized Studies—of Interventions (ROBINS‐I) tool was used in this study to assess the risk of bias for the included nonrandomized studies. The risk of bias was systematically evaluated from several aspects, including study population selection, exposure and outcome measurement, control of confounding factors, completeness of follow‐up, and data reporting. Quality assessment was independently completed by five researchers. In case of disagreement, the final decision was made through discussion and consensus. The results of each evaluation were used as an important basis for the subsequent sensitivity analyses and interpretation of results.

### 2.6. Statistical Analysis

In this study, the OR with its 95% CI was used as the effect measure. The DerSimonian–Laird random‐effects model was used for pooled analysis. Heterogeneity was assessed by the *I* ^2^ statistic and *Q* test, and sensitivity analyses were performed to test the robustness of the main results. Subgroup analyses were conducted to explore the sources of heterogeneity. Publication bias was assessed by funnel plots and Egger’s test. All statistical analyses were performed using R software, and Zotero was used for reference management. To further explore the potential sources of heterogeneity, meta‐regression analyses were conducted separately within genotype subgroups with sufficient studies (KPC and OXA‐48–like). Study‐level moderators included mean age, study design (prospective vs. retrospective cohort), sample size, follow‐up duration, and year of publication. For categorical moderators (e.g., study design), coefficients represent the effect relative to the reference category. Meta‐regression was not performed for other genotype subgroups due to the limited number of included studies.

## 3. Results

### 3.1. Literature Screening and Inclusion Process

**Figure 1:**
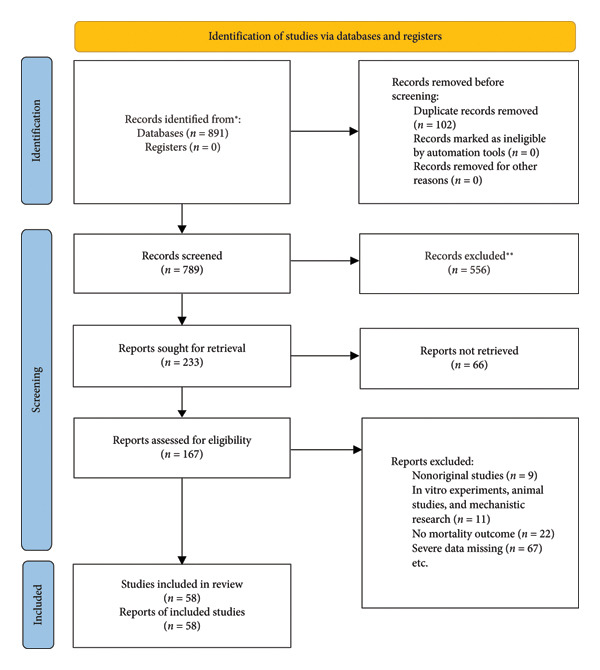
PRISMA flow diagram.

A total of 891 studies were retrieved in this study, and 789 studies remained after removing duplicates. After screening titles and abstracts, 233 studies entered the full‐text evaluation stage, of which 66 were excluded due to unavailable full text. Finally, 58 studies meeting the inclusion and exclusion criteria were included. The specific literature search strategy and screening process are shown in Figure 1 and Supporting Table 1.

### 3.2. Basic Characteristics of Included Studies and Patients

A total of 58 original studies were included in this meta‐analysis, covering multiple countries and regions such as China, Turkey, Italy, India, Brazil, Malaysia, Spain, Romania, Greece, Colombia, Saudi Arabia, the United States, and South Africa, demonstrating strong international representation. The study types included retrospective cohort studies (RCSs), prospective cohort studies (PCSs), and CCS, with sample sizes of individual studies ranging from 17 to 335 cases.

The patient populations included in each study were mainly from ICUs and general wards, with some involving high‐risk groups such as neonatal ICUs (NICUs) or organ transplant recipients. The average age of patients varied widely, including neonates, children, and elderly populations. Most follow‐up periods were 14 days, 28 days, or 30 days, with the longest follow‐up up to 306 days in some studies.

This study systematically summarized and grouped the major molecular genotypes of CRKP (KPC, NDM, OXA‐48–like, IMP, VIM, etc.). Among them, KPC and OXA‐48–like were the most commonly reported resistance genotypes, and there were also a certain number of NDM, IMP, and VIM genotypes. There was significant heterogeneity in the distribution of different genotypes across regions; for example, KPC was predominant in China, OXA‐48–like in Turkey and some European regions, and NDM in India and Brazil.

All included studies reported all‐cause mortality outcomes and provided OR with 95% CIs. Table 1 provides detailed information on each study, including the first author, year of publication, country, study design, sample size, molecular genotype, mean age, ward type, follow‐up period, number of deaths, and OR (95% CI).

**Table 1:** Inclusion of studies and basic characteristics of patients.

First author	Year	Country	Study design	Sample size	Genotype	Mean age	(ICU/GW)	Follow‐up (day)	All‐cause deaths	OR, 95% CI
Chen Y	2024	China	RCS	45	KPC	46	GW	30	37	1.23 (0.23–6.56)
Cheng Y	2024	China	RCS	50	KPC	75	ICU/GW	28	23	3.62 (1.66–7.89)
Kurt AF	2024	Turkey	RCS	230	OXA‐48–like	63.1	ICU/GW	30	160	3.54 (1.77–7.08)
Ma JY	2024	China	RCS	108	KPC	< 1	ICU、NICU/GW	90	27	0.90 (0.26–3.12)
Antochevis LC	2025	Brazil	PCS	194	NDM	NA	ICU/GW	28	331	1.38 (0.87–2.18)
Ali Mert	2024	Turkey	RCS	106	OXA‐48–like	55	ICU/GW	30	35	0.24 (0.07–0.79)
HUANG Yaxuan	2024	China	RCS	40	KPC	69	ICU	14	20	4.50 (1.00–20.00)
Lau MY	2021	Malaysia	RCS	70	OXA‐48–like	49.55	ICU/GW	14	26	2.50 (1.10–5.60)
Lima Rodríguez O	2021	Spain	RCS	88	OXA‐48–like	73.5	ICU/GW	14	26	2.20 (1.13–4.26)
Zhang N	2022	China	RCS	152	KPC	61.5	ICU、NICU/GW	30	30	3.51 (2.29–5.97)
Cox PB	2025	Singapore/USA	RCS	125	KPC	64	ICU/GW	14	57	5.20 (1.50–25.50)
Li JY	2024	China	RCS	231	KPC	52.3	ICU/GW	30	81	8.36 (1.10–23.27)
Jen‐Yu Hsu	2021	China Taiwan	CCS	36	KPC	66.8	GW	28	19	5.43 (1.63–18.06)
Del Rio A	2022	Italy	RCS	381	OXA‐48–like	70	ICU	14	42	5.39 (2.42–12.00)
Lee N‐Y	2020	China Taiwan	RCS	114	OXA‐48–like	69	ICU/GW	30	40	1.71 (0.61–4.85)
Di Domenico EG	2020	Italy	RCS	86	KPC	71	GW	30	19	6.30 (1.78–19.24)
Huang Po‐Han	2022	China Taiwan	CCS	52	KPC	65	GW	30	17	1.17 (0.05–3.62)
Sharma S	2022	India	RCS	43	OXA‐48–like	< 1	NICU	14	12	36.00 (2.70–480.10)
Chen I‐Ren	2021	China Taiwan	RCS	44	KPC	76.5	GW	14	13	8.87 (1.66–11.26)
Díaz A	2016	Colombia	RCS	34	KPC	< 1	NICU	14	13	2.22 (0.95–5.18)
Wang Z	2018	China	CCS	48	KPC	67.7	GW	14	23	11.90 (4.70–21.70)
Katsiari M	2015	Greece	PCS	32	KPC	63.5	ICU	14	23	0.81 (0.13–5.01)
Abi Manesh	2023	India	PCS	181	OXA‐48–like	43	ICU	30	90	4.63 (2.22–9.66)
Dongmei Lv	2022	China	RCS	103	KPC	58	ICU	30	31	3.12 (1.08–8.98)
Dragos S. Lazar	2024	Romania	RCS	162	OXA‐48–like	67.4	ICU/GW	14	50	5.98 (1.59–22.36)
Yufei Zhang	2024	China	RCS	123	KPC	82	ICU/GW	14	49	5.87 (1.32–26.13)
Uğur Önal	2023	Turkey	RCS	42	OXA‐48–like	59.4	ICU	30	23	17.44 (1.19–256.28)
Lumbreras‐Iglesias	2024	Spain	RCS	76	OXA‐48–like	66.7	ICU/GW	30	24	4.50 (1.00–20.00)
Markovska	2024	Bulgaria	RCS	44	OXA‐48–like	68.4	ICU/GW	14	11	3.60 (0.48–27.11)
Sotgiu	2018	Italy	PCS	46	KPC	69.4	ICU/GW	30	23	2.10 (0.63–7.01)
Liu	2021	China Taiwan	RCS	89	KPC	75.6	ICU/GW	30	46	3.44 (1.37–8.63)
Jiao	2015	China	RCS	30	KPC	59.8	ICU/GW	30	10	2.16 (0.11–5.39)
Mammina	2013	Italy	RCS	31	KPC	53.6	ICU	23	13	2.01 (0.75–5.36)
Cheng	2023	China	RCS	98	KPC	61.58	ICU	30	32	1.19 (0.06–3.67)
Xiang	2024	China	RCS	311	KPC	63.6	ICU	14	33	1.89 (1.06–3.38)
Gaspar	2022	Brazil	RCS	26	KPC	57.8	ICU/GW	14	8	10.5 (1.50–23.67)
Gregory	2010	Puerto Rico	CCS	26	KPC	70	ICU/GW	14	NA	2.20 (0.60–7.90)
Li M	2025	China	PCS	29	KPC	48	GW	28	12	9.87 (3.68–26.42)
Xue Y	2025	China	RCS	335	IMP	56	ICU/GW	30	36	14.52 (1.94–108.41)
Zhao CF	2025	China	PCS	238	KPC	58	ICU	28	43	12.87 (5.72–26.62)
Onorato L	2022	Italy	RCS	174	KPC	60.8	ICU/GW	90	62	2.71 (1.22–6.07)
Verma A	2022	India	RCS	49	NDM	< 1	ICU/GW	14	31	0.51 (0.13–2.02)
Qiu M	2025	China	RCS	177	KPC	49	ICU/GW	14	22	1.76 (0.71–4.35)
Amir Mohammad Ali Tabrizi	2018	Iran	RCS	53	VIM	40	ICU	14	4	5.49 (2.43–12.37)
Melinte V	2025	Romania	RCS	91	KPC	63.7	ICU/GW	14	16	4.80 (3.50–23.30)
Cienfuegos‐Gallet AV	2019	Colombia	CCS	37	KPC	64	ICU/GW	30	16	1.44 (0.24–4.84)
Abu Jaber AMR	2024	Saudi Arabia	RCS	114	OXA‐48–like	71	ICU/GW	30	67	0.43 (0.20–0.93)
Machuca I	2019	Spain	RCS	100	KPC	64	ICU/GW	30	31	1.56 (0.69–3.33)
Yao Chen	2025	China	RCS	209	KPC	67.3	ICU/GW	28	30	2.54 (1.38–4.69)
Mouloudi E	2014	Greece	CCS	17	KPC	54	ICU	30	14	15.50 (2.00–108.00)
Büyüktuna SA	2020	Turkey	RCS	32	OXA‐48–like	74	ICU	28	23	5.15 (1.84–14.4)
Kong ZX	2022	Malaysia	RCS	245	OXA‐48–like	54.9	ICU/GW	14	56	0.92 (0.37–2.31)
Wang J	2025	China	RCS	45	KPC	40	GW	30	20	4.81 (1.21–19.05)
Rojas LJ	2017	USA	RCS	238	KPC	67	GW	30	NA	3.48 (1.73–6.57)
Magobo RE	2023	South Africa	RCS	26	NDM	7	NICU	14	14	0.64 (0.09–4.58)
Capone A	2013	Italy	RCS	97	KPC	69	ICU/GW	14	25	2.62 (0.13–12.55)
Gomez‐Simmonds	2015	USA	RCS	29	KPC	58	ICU/GW	30	12	8.30 (0.70–20.40)
van Duin D	2015	USA	PCS	157	KPC	72	ICU/GW	306	16	5.82 (1.47–28.50)

Abbreviations: CCS = case–control study, GW = general ward, ICU = intensive care unit, NICU = neonatal intensive care unit, PCS = prospective cohort study, RCS = retrospective cohort study.

Across the 58 included studies, the most frequently reported genotype was KPC, accounting for 34 studies, followed by OXA‐48–like (16 studies), NDM (3 studies), IMP (1 study), and VIM (1 study). KPC genotypes were predominant in China, as well as several studies from Europe and the United States. OXA‐48–like genotypes were mainly reported in Turkey, Mediterranean countries, and some studies from China and Malaysia. NDM was primarily reported in India, Brazil, and South Africa, while IMP and VIM genotypes were relatively rare and scattered. The detailed genotype distribution by country and region is shown in Table 1. This highlights the clear geographic heterogeneity in the genotypic spectrum of CRKP (see Table 1 for details).

### 3.3. Quality Assessment and Risk of Bias

**Figure 2:**
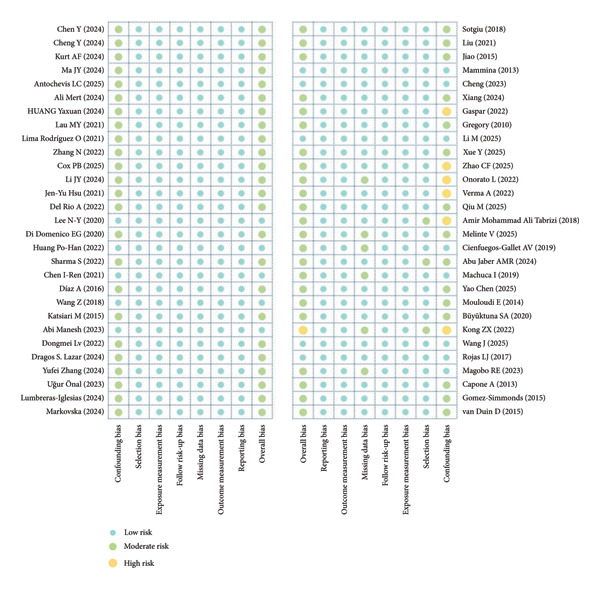
ROBINS‐I risk of bias assessment.

The ROBINS‐I tool was used in this study to systematically assess the risk of bias for all 58 included original studies, covering major domains such as confounding bias, selection bias, exposure measurement bias, follow‐up bias, missing data bias, outcome measurement bias, and reporting bias. The results showed that most studies had a low risk of bias in selection, exposure measurement, follow‐up, and outcome measurement. However, there was generally some insufficiency in the control of confounding factors, with some studies lacking adequate adjustment for covariates or having missing data, resulting in an overall moderate risk of bias, with a few studies classified as high risk. The risk distribution across ROBINS‐I domains for the included studies is shown in Figure 2, and the detailed results for each item are provided in Supporting Table 2.

### 3.4. Meta‐Analysis Results of Overall Mortality Risk for Different CRKP Genotypes

**Figure 3:**
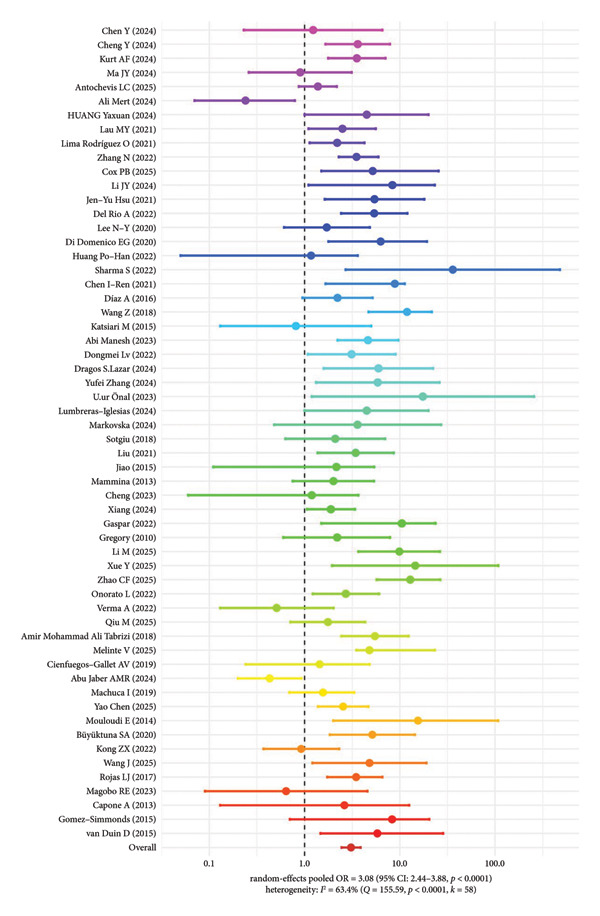
Overall mortality risk of different CRKP genotypes.

According to the random‐effects model, a pooled analysis of 58 studies showed that the combined OR for all‐cause mortality in patients infected with different molecular genotypes of CRKP was 3.08 (95% CI: 2.44–3.88, *p* < 0.0001), indicating that infection with major CRKP genotypes was significantly associated with a higher risk of death (Figure 3). The heterogeneity of the pooled analysis was *I* ^2^ = 63.4% (*Q* = 155.59, *p* < 0.0001), indicating moderate to substantial heterogeneity among the studies.

### 3.5. Subgroup Analysis

#### 3.5.1. Mortality Risk Analysis of Patients With KPC Genotype

**Figure 4:**
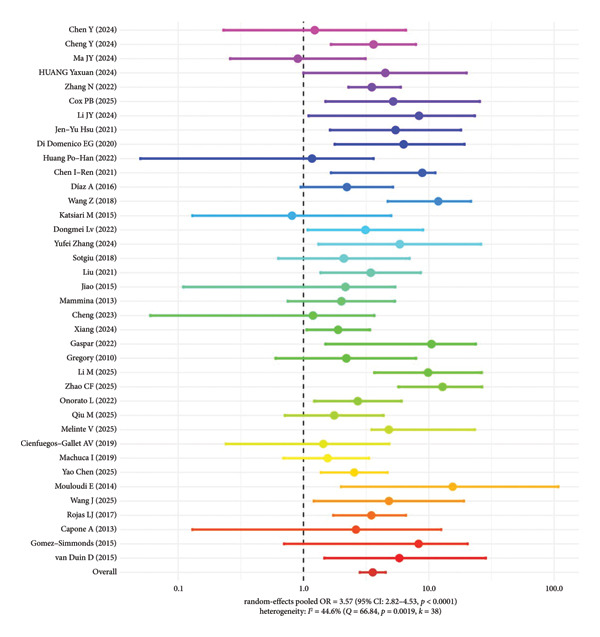
Mortality risk in patients infected with KPC genotype CRKP.

A total of 38 studies were included in the meta‐analysis for patients infected with the KPC genotype CRKP. The pooled results showed that KPC genotype CRKP infection significantly increased the risk of all‐cause mortality, with a combined OR of 3.57 (95% CI: 2.82–4.53, *p* < 0.0001) and moderate heterogeneity (*I* ^2^ = 44.6%) (Figure 4).

##### 3.5.1.1. Subgroup Analysis of KPC Genotype in China

In China, 21 KPC genotype‐related studies were included. The pooled analysis showed that the OR for mortality in patients with KPC genotype CRKP infection in China was 3.85 (95% CI: 2.74–5.42, *p* < 0.0001), with heterogeneity *I* ^2^ = 57.1%. This suggests a more pronounced impact of KPC genotype infection on mortality risk in China, but there was some variability among study results (Table 2).

**Table 2:** Subgroup analysis of mortality risk and heterogeneity.

Group	*k*	OR (95% CI)	*p* value	*I* ^2^	*Q*	*p* value for *Q* test
KPC (other countries)	17	3.04 (2.32–4.00)	< 0.0001	13.20%	18.44	0.2987
KPC (China)	21	3.85 (2.74–5.42)	< 0.0001	57.10%	46.62	0.0007
KPC genotype	38	3.57 (2.82–4.53)	< 0.0001	44.60%	66.84	0.0019
OXA‐48–like genotype	15	2.57 (1.47–4.50)	0.001	75.40%	57.02	< 0.0001
Other resistant genotypes	5	2.00 (0.66–6.07)	0.2235	76.40%	16.95	0.002

##### 3.5.1.2. Subgroup Analysis of KPC Genotype in Other Countries and Regions

In countries and regions outside China, 17 KPC genotype‐related studies were included. The pooled OR was 3.04 (95% CI: 2.32–4.00, *p* < 0.0001), with low heterogeneity (*I* ^2^ = 13.2%), and the results among studies were relatively consistent (Table 2).

#### 3.5.2. Mortality Risk Analysis of Patients With OXA‐48‐Like Genotype

**Figure 5:**
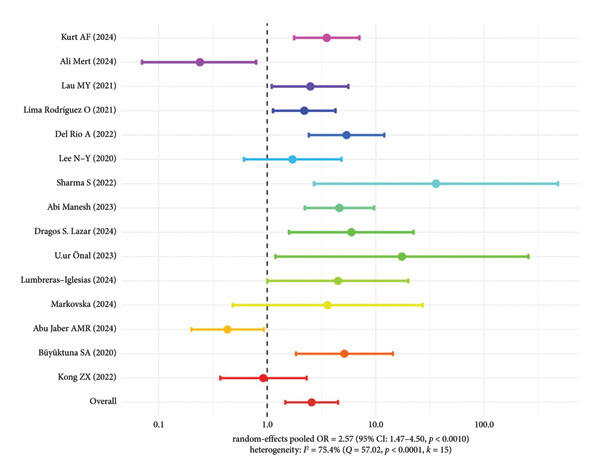
Mortality risk in patients infected with OXA‐48–like genotype CRKP.

For patients infected with the OXA‐48–like genotype CRKP, a total of 15 studies were included. The pooled OR was 2.57 (95% CI: 1.47–4.50, *p* = 0.0010), with high heterogeneity (*I* ^2^ = 75.4%). The results suggest that OXA‐48–like genotype infection also increases the risk of mortality, but there is considerable heterogeneity among studies, which needs further explanation considering specific regions and patient characteristics (Figure 5).

#### 3.5.3. Mortality Risk Analysis of Other Resistant Genotypes (NDM, IMP, VIM, etc.)

**Figure 6:**
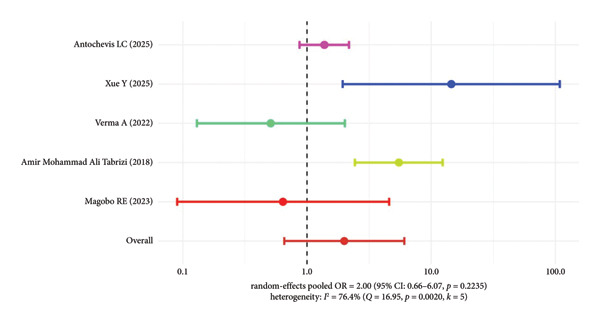
Mortality risk in patients infected with other resistant genotypes of CRKP.

For other resistant genotypes such as NDM, IMP, and VIM, due to the small number of included studies, pooled analysis was not conducted for each genotype separately. The pooled OR of five studies was 2.00 (95% CI: 0.66–6.07, *p* = 0.2235), with high heterogeneity (*I* ^2^ = 76.4%), indicating a trend toward increased mortality risk with these genotypes, but the statistical significance was not reached, and there was considerable inconsistency among study results (Figure 6).

#### 3.5.4. Summary of Subgroup Analysis

In summary, the results of subgroup analyses indicate that KPC genotype CRKP infection significantly increases the risk of mortality in both China and other countries and regions, with a slightly higher pooled effect size and greater heterogeneity in China. OXA‐48–like and other genotypes also show a trend of increased risk, but the robustness and consistency of the evidence need to be further enhanced. Regional epidemiological features, patient composition, and treatment differences may have an important impact on outcomes.

### 3.6. Meta‐Regression Analyses Within Genotype Subgroups

Meta‐regression analyses within the KPC subgroup (*n* = 38) showed that study design explained 38% of the between‐study heterogeneity (*R* ^2^ = 38.0%), but the effect was not statistically significant. Mean age, sample size, follow‐up duration, and year of publication were not significantly associated with mortality risk and explained little or none of the observed heterogeneity (all *p* > 0.05, *R* ^2^ ≈ 0%). In the OXA‐48–like subgroup (*n* = 15), none of the examined moderators, including age, study design, sample size, follow‐up duration, and year of publication, were significantly associated with mortality risk, nor did they explain the substantial heterogeneity observed (all *R* ^2^ = 0.00%, residual *I* ^2^ > 77%). The detailed results are presented in Table 3.

**Table 3:** Meta‐regression results for study‐level moderators within KPC and OXA‐48–like genotype subgroups.

Genotype	Moderator	Coefficient (*β*)	95% CI	*p* value	*R* ^2^ (%)	Residual *I* ^2^ (%)
KPC	Age	0.0114	−0.0025, 0.0252	0.107	7.36	44.4
	StudyDesign PCS	0.178	−0.691, 1.047	0.688	38.04	34.7
	StudyDesign RCS	−0.456	−1.120, 0.209	0.179	—	—
	Sample size	−0.0007	−0.0037, 0.0022	0.628	0	46.6
	Follow‐up duration	−0.0003	−0.0061, 0.0055	0.927	0	47.7
	Year of publication	0.0216	−0.0388, 0.0820	0.483	0	47

OXA‐48–like	Age	−0.0219	−0.0614, 0.0177	0.279	0	78.3
	StudyDesign RCS	−0.636	−2.7414, 1.4694	0.554	0	77.9
	Sample size	−0.0001	−0.0063, 0.0060	0.966	0	79.2
	Follow‐up duration	−0.0319	−0.1046, 0.0409	0.39	0	77.8
	Year of publication	−0.1219	−0.5069, 0.2632	0.535	0	78.3

*Note:* *R* ^2^ and residual *I* ^2^ refer to the overall model for each moderator. For categorical moderators (e.g., study design), coefficients represent the effect relative to the reference category.

### 3.7. Sensitivity Analysis

**Figure 7:**
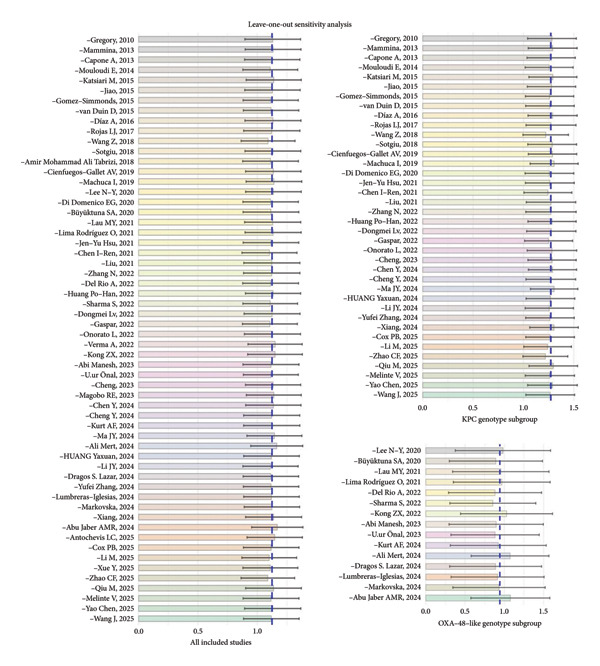
Leave‐one‐out sensitivity analysis of all included studies.

To test the robustness of the main pooled analysis results, a leave‐one‐out sensitivity analysis was conducted for all studies, as well as for the KPC genotype and OXA‐48–like genotype subgroups. The results showed that, for the overall analysis, the KPC genotype subgroup, and the OXA‐48–like genotype subgroup, the combined effect size did not change substantially after sequentially removing any single study. This indicates that the overall meta‐analysis results are robust and reliable (see Figure 7). No single study was observed to have a decisive impact on the overall conclusions, which further strengthens the evidence provided by this study.

### 3.8. Publication Bias Analysis

**Figure 8:**
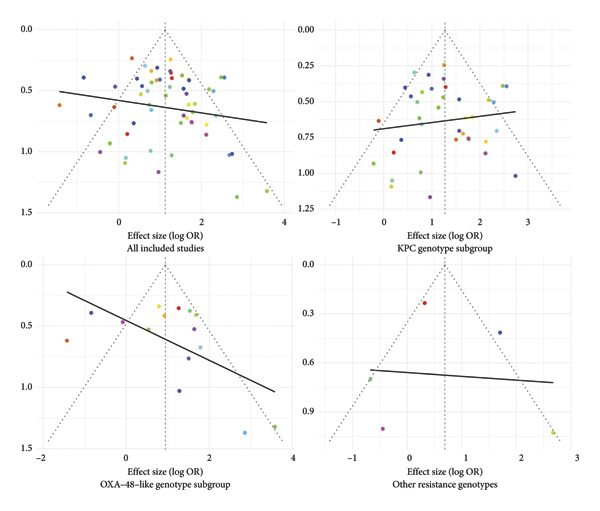
Funnel plot of all included studies.

To assess the possibility of publication bias, funnel plot visualization and statistical tests were conducted for all included studies, as well as for the KPC genotype, OXA‐48–like genotype, and other resistant genotypes. The results showed that no obvious asymmetry was observed in the funnel plots of each subgroup (Figure 8).

Further statistical assessments using Egger’s test and Begg’s test were performed for all studies, the KPC genotype, and the OXA‐48–like genotype. For all studies: Egger’s test *z* = 0.8588, *p* = 0.3904; Begg’s test tau = 0.0666, *p* = 0.4606. For the KPC genotype: Egger’s test *z* = −0.2801, *p* = 0.7794; Begg’s test tau = 0.0128, *p* = 0.9205. For the OXA‐48–like genotype: Egger’s test *z* = 1.7608, *p* = 0.0783; Begg’s test tau = 0.1238, *p* = 0.5590. All of these *p* values were greater than 0.05, indicating that no significant publication bias was detected. Because of the limited number of studies included in the other resistant genotype group, Egger’s and Begg’s tests were not conducted for this subgroup. The risk of publication bias in the studies included in this meta‐analysis was low, and the main analysis results are robust and interpretable.

### 3.9. GRADE Quality of Evidence

In this study, the quality of evidence for the main outcomes was assessed using the GRADE system. The quality of evidence for the meta‐analysis of the association between KPC genotype CRKP infection and mortality risk was rated as moderate, mainly limited by the fact that some included studies were retrospective cohorts, had certain risks of bias, and exhibited moderate heterogeneity. The quality of evidence related to OXA‐48–like and NDM/IMP genotypes was relatively low, mainly due to limited sample sizes, higher inconsistency of results, and nonuniform outcome measurements. Detailed GRADE assessment results are provided in Supporting Table 3.

## 4. Discussion

### 4.1. Main Findings and Comparison With Literature

This meta‐analysis demonstrates that CRKP infection, especially those caused by KPC and OXA‐48–like genotypes, is significantly associated with increased all‐cause mortality. The effect is particularly pronounced in China, possibly reflecting the predominance of hypervirulent KPC‐ST11 clones and region‐specific healthcare practices. Our findings are in line with previous large‐scale studies, which have consistently reported KPC as the leading genotype linked to adverse outcomes. By systematically comparing major genotypes and including regional subgroup analysis, this study adds quantitative evidence on the genotype‐ and region‐specific mortality risks. Notably, although NDM, IMP, and VIM genotypes also showed a trend toward higher mortality, the limited number of studies and substantial heterogeneity warrant cautious interpretation. Persistent moderate‐to‐high heterogeneity—only partially explained by molecular genotype and regional differences—underscores the complexity of clinical and epidemiological factors influencing outcomes. These results highlight the need for more standardized research and further exploration of unmeasured confounders in future studies.

### 4.2. Clinical and Public Health Significance

#### 4.2.1. Clinical Application of Risk Stratification for High‐Risk Genotypes

There are significant differences in mortality risk among patients infected with different molecular genotypes of CRKP, with KPC and OXA‐48–like genotypes conferring particularly high mortality risks. These findings underscore the necessity of risk stratification management in clinical practice for CRKP‐infected patients based on their resistance genotypes. Multiple studies have confirmed that various resistance mechanisms and genotypes not only determine antibiotic susceptibility and treatment difficulty, but are also closely linked to clinical outcomes. KPC genotype CRKP is globally prevalent, often associated with higher virulence and poor prognosis, and is a major pathogen in hospital‐acquired infections in China as well as in Europe and North America [10–35]. In recent years, the OXA‐48–like genotype has become increasingly predominant in Mediterranean countries, Turkey, and western China, with related infections showing rising mortality rates year by year [13, 36–40].

Effective risk stratification relies on early identification of high‐risk genotypes, especially among critically ill patients, those with multiple comorbidities, ICU patients, and other high‐risk groups. Studies indicate that KPC and OXA‐48–like CRKP infections are more common in patients with histories of invasive procedures, prolonged hospitalization, and immunosuppression, resulting in a higher likelihood of invasive infections, organ dysfunction, and increased mortality compared to other genotypes [11, 13, 41, 42]. Therefore, clinical management should incorporate stratified early warning and multidisciplinary intervention based on molecular typing and patient severity, with an emphasis on enhanced infection control and individualized treatment.

The regional characteristics of OXA‐48–like genotype prevalence, especially in western China and “Belt and Road” countries, present new challenges for infection control. OXA‐48–like CRKP often demonstrates covert transmission and a broad resistance spectrum and can rapidly cluster during outbreaks, heightening nosocomial infection risk [13, 36, 42]. This study systematically evaluated the mortality risk associated with KPC and OXA‐48–like genotypes in China, providing direct evidence for precision interventions and region‐specific prevention and control measures. Early identification of high‐risk clones through surveillance and molecular tracing, together with real‐time risk alerts for key departments (such as ICUs) and high‐risk procedures, may help reduce cluster outbreaks and nosocomial transmission [12, 36, 43].

Genotype‐based clinical decision‐making has also been proposed: For KPC and OXA‐48–like CRKP infections, early initiation of effective combination antibiotic therapy, strengthened infection control, and dynamic resistance monitoring can improve prognosis, reduce mortality, and lessen medical burden [10, 12, 36, 43].

Incorporating high‐risk genotypes into the CRKP infection risk stratification system enhances early warning, optimizes patient management, and enables more precise infection prevention and control. This approach not only is applicable in China but also offers valuable experience for precision control of CRKP infection in high‐incidence regions globally [13, 41, 43–62].

#### 4.2.2. Optimization of Antimicrobial Management and Clinical Decision‐Making

Significant differences in antibiotic susceptibility exist among CRKP strains with different resistance genotypes. The rational use of molecular typing information can thus optimize both antimicrobial management and clinical decision‐making. Studies have shown that patients infected with high‐risk genotypes such as KPC, OXA‐48–like, and NDM have poorer prognoses, indicating that traditional empirical treatments are often inadequate. Therefore, individualized and precision anti‐infective interventions based on genotype are increasingly required in clinical practice [10–12, 42, 47–67].

Molecular typing provides an evidence‐based foundation for selecting effective antibiotics. New β‐lactamase inhibitors, such as ceftazidime–avibactam and meropenem–vaborbactam, have demonstrated good activity against KPC genotypes and are associated with improved outcomes in critically ill patients [10, 12, 42]. For high‐risk genotypes like NDM and OXA‐48–like, conventional β‐lactam antibiotics are less effective, necessitating the use of last‐resort regimens such as tigecycline, colistin, fosfomycin, or combination therapies. Drug accessibility and the potential for resistance development should also be considered in clinical decisions, particularly in resource‐limited settings [11–13, 36, 43, 56–71].

Antibiotic management guided by molecular typing helps avoid indiscriminate use of broad‐spectrum antibiotics, reduces selection pressure, and enhances infection control. Multicenter studies in China and internationally confirm that integrating typing information into prescribing processes increases rationality and targeting of therapy, decreases unnecessary use of high‐intensity agents, and reduces adverse events and resistance transmission [10, 41–43]. Rapid feedback mechanisms based on typing, especially in high‐risk settings such as ICUs, hemodialysis, and transplant units, facilitate the dynamic adjustment of treatment strategies and perioperative management, achieving precise “population–pathogen–medication” interventions.

Furthermore, Chinese guidelines and expert consensus recommend timely molecular typing for critically ill and high‐risk CRKP patients, with antibiotic selection and combination strategies adjusted accordingly to improve clinical response and prognosis [10–13, 36, 41]. Genotype‐guided optimization not only informs individualized treatment but also supports evidence‐based policy and regional antimicrobial stewardship initiatives.

#### 4.2.3. Hospital Infection Control, Multicenter Collaboration, and Policy Optimization

This study provides important evidence for optimizing hospital infection control, strengthening multicenter collaboration, and informing policy decisions regarding resource allocation. With the rising prevalence of multidrug‐resistant bacteria such as CRKP, challenges including hospital outbreaks, cross‐facility transmission, and regional epidemics highlight the urgent need for more coordinated and evidence‐based infection control systems [37, 38].

Routine molecular typing, particularly in high‐risk departments (ICU, hematology, transplantation, critical care), enables early identification and targeted management of colonized or infected patients, supporting timely outbreak response and individualized interventions [8, 37].

Multicenter collaboration and regional data sharing further enhance surveillance, facilitate early warning, and support integrated responses to transmission across facilities. Regional infection control networks and partnerships among hospitals, public health authorities, and laboratories can optimize coverage and effectiveness in curbing the spread of resistant bacteria [37, 38].

Finally, our findings provide a scientific foundation for policymakers to rationally allocate infection control resources, prioritize high‐risk areas, and develop policies supporting capacity building for molecular typing and big data infrastructure. Such measures will help achieve precision deployment of infection control, improve return on investment, and reduce both healthcare and societal burdens [37, 38].

### 4.3. Mechanistic Explanations and Scientific Hypotheses

The high mortality associated with major CRKP genotypes such as KPC, OXA‐48–like, and NDM results from the convergence of multidrug resistance and enhanced virulence. KPC strains, for example, harbor the *b* *l* *a*_KPC_ gene and often cocarry ESBLs, conferring resistance to most β‐lactams and contributing to the spread of extensively drug‐resistant phenotypes (XDR)^[77]^. OXA‐48–like and NDM genotypes confer resistance through carbapenemase production, porin loss, and metallo‐β‐lactamase activity [39, 40]. Recent research also implicates regulatory elements such as small RNAs (e.g., sRNA207) in enhancing biofilm formation and adaptability, providing potential targets for novel anti‐infective strategies [28]. Additionally, hypermucoviscosity, siderophore production, and biofilm‐forming ability further augment the pathogenicity and clinical impact of these strains [4, 40].

Differences in clinical outcomes among patients with various CRKP genotypes are shaped by a combination of microbial and host factors, including resistance profiles, virulence traits, underlying comorbidities, and exposure risks such as ICU admission or invasive procedures. In China, the high prevalence of hypervirulent KPC‐ST11 clones and the emergence of NDM‐5 and OXA‐48–like variants have driven increased mortality and complicated infection control efforts [4, 38, 39]. The intestine serves as a key reservoir for colonization and subsequent invasive infection, especially in critically ill patients [38].

Integrating current molecular research, it is hypothesized that synergistic mechanisms—including enzyme activity, membrane alterations, and regulatory RNAs—drive the dual phenotype of resistance and virulence in high‐risk CRKP genotypes. Continued investigation of genotype evolution, host–pathogen interactions, and new molecular targets is essential for developing precision prevention and individualized therapies in the future [4, 38–40].

### 4.4. Analysis of Noncarbapenemase‐Producing CRKP

This study primarily focused on CRKP isolates with clearly defined carbapenemase genotypes, such as KPC, OXA‐48–like, NDM, IMP, and VIM, as these were the main types reported in the included studies. However, noncarbapenemase‐producing CRKP strains—which acquire carbapenem resistance mainly through mechanisms such as porin loss and ESBL production—were seldom reported as independent groups in the original literature. Due to the limited availability of detailed clinical and prognostic data for these strains, we were unable to perform subgroup analyses to compare their mortality risk. Therefore, the current findings regarding genotype‐specific mortality should be interpreted in this context, and further research is needed to elucidate the clinical outcomes and management of noncarbapenemase‐producing CRKP.

### 4.5. Limitations

There are several limitations in this study that should be considered when interpreting the results.

Most included studies were retrospective cohorts or single‐center designs, which may introduce selection and information bias, as well as subjective variation in case identification, exposure ascertainment, and outcome assessment. In addition, some original studies had limited sample sizes, particularly for nonmajor genotypes such as NDM, IMP, and VIM, resulting in insufficient statistical power in subgroup analyses and limiting the robustness and generalizability of the conclusions. There was also inconsistency in genotyping methods, outcome definitions (e.g., in‐hospital mortality, 30‐day mortality, all‐cause mortality), and follow‐up durations across studies, which may have affected comparability and the accuracy of pooled estimates.

Although comprehensive subgroup and meta‐regression analyses were conducted by genotype, region, and other study‐level characteristics, moderate‐to‐high heterogeneity persisted in several pooled results. None of the tested study‐level moderators, including mean age, study design, sample size, follow‐up duration, and year of publication, could explain the observed heterogeneity within the main genotype subgroups. This suggests the presence of unmeasured confounding factors—such as differences in treatment regimens, spectrum of underlying diseases, proportion of critically ill patients, and occurrence of nosocomial outbreaks—which could not be fully accounted for by available data.

In particular, variations in antimicrobial therapy (e.g., choice of agents, timing, use of combination versus monotherapy, and access to novel drugs) are a major confounding factor affecting patient mortality. Most included studies did not provide detailed or standardized data on treatment regimens, and thus, we were unable to adjust for these differences in our analysis. As a result, the association between genotype and mortality risk reported in this study may be partially influenced by unmeasured confounding from therapeutic strategies.

Additionally, some studies lacked detailed reporting of confounder adjustment and did not clearly distinguish infection types (e.g., colonization versus infection, bloodstream versus pulmonary infections), resulting in further limitations in risk attribution at the individual level. Finally, some mechanistic explanations and scientific inferences proposed in this study require further validation through experimental research and prospective multicenter cohort studies.

Due to the limited number of studies reporting noncarbapenemase‐producing CRKP as separate groups, this study was unable to analyze the mortality risk for these strains, highlighting the need for further investigation in future research.

### 4.6. Prospects and Recommendations

It is recommended that future research conduct multicenter, prospective cohort, or randomized controlled studies to improve the quality and representativeness of evidence. Efforts should be made to strengthen CRKP molecular typing detection and the standardization of epidemiological data, thereby promoting data sharing and joint analyses. Further in‐depth studies are needed to explore molecular mechanisms such as the convergence of resistance and hypervirulence and to elucidate the intrinsic links between different genotypes and clinical outcomes. Meanwhile, it is suggested that risk stratification management of high‐risk genotypes should be incorporated into routine hospital infection control and clinical decision‐making processes, providing a solid foundation for individualized treatment and public health policy development.

## 5. Conclusion

This meta‐analysis systematically evaluated the mortality risk of patients infected with different molecular genotypes of CRKP. The results showed that infections with high‐risk genotypes such as KPC and OXA‐48–like CRKP were significantly associated with increased mortality risk, and the pooled effect value for KPC genotype in China was higher than that in other countries and regions. Sensitivity and subgroup analysis results indicated that these conclusions are relatively robust.

This study not only supplements and refines the evidence base for mortality risk of CRKP infections with different resistance genotypes, but also provides a data foundation for the risk stratification of high‐risk patients, individualized treatment decisions, and optimization of regional prevention and control strategies.

In the future, it is necessary to further strengthen multicenter cohort and mechanistic studies, improve genotyping diagnosis, data standardization, and collaborative prevention and control systems, and continuously improve the management of CRKP infections. Based on our findings, we specifically recommend that hospitals incorporate the molecular typing of CRKP into routine clinical diagnostics and infection risk stratification, particularly for high‐risk departments such as ICUs and transplant units. Regionally tailored surveillance and early warning systems should be established to rapidly identify and contain outbreaks of high‐risk genotypes (KPC, OXA‐48–like). Clinical management guidelines should be updated to support the genotype‐guided optimization of antimicrobial therapy, ensuring the timely initiation of effective combination regimens for high‐risk patients. These measures will facilitate precise infection control and improve patient outcomes.

## Disclosure

All authors have read and approved the final manuscript and agree to be accountable for all aspects of the work.

## Conflicts of Interest

The authors declare no conflicts of interest.

## Author Contributions

Qiongfang Zhang: conceptualization, methodology, data curation, formal analysis, writing–original draft, and project administration.

Ze Fang: supervision, funding acquisition, methodology, and review and editing.

Bo Fang: data collection, investigation, resources, and review and editing.

Hailing Zeng: validation, data analysis, literature search, and review and editing.

Jiaojiao Xu: literature search, data validation, and review and editing.

## Funding

No funding was received for this study.

## Supporting Information

Additional supporting information can be found online in the Supporting Information section.

## Supporting information


**Supporting Information 1** PRISMA_2020_checklist: Completed PRISMA 2020 checklist for systematic reviews and meta‐analyses.

**Supporting Information 2** Supporting Table 1: Detailed search strategies used for all included databases.

**Supporting Information 3** Supporting Table 2: ROBINS‐I risk of bias assessment for nonrandomized studies.

**Supporting Information 4** Supporting Table 3: GRADE quality of evidence assessment for the main outcomes.

## Data Availability

Research data are not shared.
