# Supplementary material for: Clinical Epidemiological Analysis of the Genotypic Spectrum and Mortality Risk in Carbapenem‐Resistant Klebsiella pneumoniae (CRKP) Infections
Source: Can J Infect Dis Med Microbiol. 2026 Jan 6;2026:1529426. doi: 10.1155/cjid/1529426 (PMC12771615; doi:10.1155/cjid/1529426)
Supplement: Supplementary file 2 — Supporting Information 2 Supporting Table 1: Detailed search strategies used for all included databases. [file CJID-2026-1529426-s002.docx]

| Supplementary Table 1. Search strategy | | |
| --- | --- | --- |
| Database | Search strategy | Results |
| PubMed | ("carbapenem-resistant Klebsiella pneumoniae"[Title/Abstract] OR "CRKP"[Title/Abstract]) AND (genotype*[Title/Abstract] OR "KPC"[Title/Abstract] OR "NDM"[Title/Abstract] OR "OXA-48"[Title/Abstract] OR "VIM"[Title/Abstract] OR "IMP"[Title/Abstract] OR "carbapenemase"[Title/Abstract]) AND (mortality[Title/Abstract] OR death[Title/Abstract] OR "fatal outcome"[Title/Abstract] OR "case fatality"[Title/Abstract]) | 227 |
| Embase | ('carbapenem resistant klebsiella pneumoniae'/exp OR 'carbapenem-resistant klebsiella pneumoniae':ab,ti OR crkp:ab,ti) AND (genotype*:ab,ti OR kpc:ab,ti OR ndm:ab,ti OR oxa-48:ab,ti OR vim:ab,ti OR imp:ab,ti OR carbapenemase*:ab,ti) AND (mortality/exp OR mortality:ab,ti OR death:ab,ti OR 'fatal outcome':ab,ti OR 'case fatality':ab,ti) | 383 |
| Web of Science | ("carbapenem-resistant Klebsiella pneumoniae" OR CRKP) AND (genotype* OR KPC OR NDM OR OXA-48 OR VIM OR IMP OR carbapenemase*) AND (mortality OR death OR "fatal outcome" OR "case fatality") | 281 |
| DATE：2025-09-09 | | |
