# Supplementary material for: Clinical Epidemiological Analysis of the Genotypic Spectrum and Mortality Risk in Carbapenem‐Resistant Klebsiella pneumoniae (CRKP) Infections
Source: Can J Infect Dis Med Microbiol. 2026 Jan 6;2026:1529426. doi: 10.1155/cjid/1529426 (PMC12771615; doi:10.1155/cjid/1529426)
Supplement: Supplementary file 3 — Supporting Information 3 Supporting Table 2: ROBINS‐I risk of bias assessment for nonrandomized studies. [file CJID-2026-1529426-s003.docx]

Supplementary Table 2

| First Author (Year) | Confounding Bias | Selection Bias | Exposure Measurement Bias | FolLow risk-up Bias | Missing Data Bias | Outcome Measurement Bias | Reporting Bias | Overall Bias |
| --- | --- | --- | --- | --- | --- | --- | --- | --- |
| Chen Y (2024) | Moderate risk | Low risk | Low risk | Low risk | Low risk | Low risk | Low risk | Moderate risk |
| Cheng Y (2024) | Moderate risk | Low risk | Low risk | Low risk | Low risk | Low risk | Low risk | Moderate risk |
| Kurt AF (2024) | Moderate risk | Low risk | Low risk | Low risk | Low risk | Low risk | Low risk | Moderate risk |
| Ma JY (2024) | Moderate risk | Low risk | Low risk | Low risk | Low risk | Low risk | Low risk | Moderate risk |
| Antochevis LC (2025) | Moderate risk | Low risk | Low risk | Low risk | Low risk | Low risk | Low risk | Moderate risk |
| Ali Mert (2024) | Moderate risk | Low risk | Low risk | Low risk | Low risk | Low risk | Low risk | Moderate risk |
| HUANG Yaxuan (2024) | Moderate risk | Low risk | Low risk | Low risk | Low risk | Low risk | Low risk | Moderate risk |
| Lau MY (2021) | Moderate risk | Low risk | Low risk | Low risk | Low risk | Low risk | Low risk | Moderate risk |
| Lima Rodríguez O (2021) | Moderate risk | Low risk | Low risk | Low risk | Low risk | Low risk | Low risk | Moderate risk |
| Zhang N (2022) | Moderate risk | Low risk | Low risk | Low risk | Low risk | Low risk | Low risk | Moderate risk |
| Cox PB (2025) | Moderate risk | Low risk | Low risk | Low risk | Low risk | Low risk | Low risk | Moderate risk |
| Li JY (2024) | Moderate risk | Low risk | Low risk | Low risk | Low risk | Low risk | Low risk | Moderate risk |
| Jen-Yu Hsu (2021) | Moderate risk | Low risk | Low risk | Low risk | Low risk | Low risk | Low risk | Moderate risk |
| Del Rio A (2022) | Moderate risk | Low risk | Low risk | Low risk | Low risk | Low risk | Low risk | Moderate risk |
| Lee N-Y (2020) | Low risk | Low risk | Low risk | Low risk | Low risk | Low risk | Low risk | Low risk |
| Di Domenico EG (2020) | Moderate risk | Low risk | Low risk | Low risk | Low risk | Low risk | Low risk | Moderate risk |
| Huang Po-Han (2022) | Low risk | Low risk | Low risk | Low risk | Low risk | Low risk | Low risk | Low risk |
| Sharma S (2022) | Moderate risk | Low risk | Low risk | Low risk | Low risk | Low risk | Low risk | Moderate risk |
| Chen I-Ren (2021) | Low risk | Low risk | Low risk | Low risk | Low risk | Low risk | Low risk | Low risk |
| Díaz A (2016) | Moderate risk | Low risk | Low risk | Low risk | Low risk | Low risk | Low risk | Moderate risk |
| Wang Z (2018) | Low risk | Low risk | Low risk | Low risk | Low risk | Low risk | Low risk | Low risk |
| Katsiari M (2015) | Moderate risk | Low risk | Low risk | Low risk | Low risk | Low risk | Low risk | Moderate risk |
| Abi Manesh (2023) | Low risk | Low risk | Low risk | Low risk | Low risk | Low risk | Low risk | Low risk |
| Dongmei Lv (2022) | Moderate risk | Low risk | Low risk | Low risk | Low risk | Low risk | Low risk | Moderate risk |
| Dragos S. Lazar (2024) | Moderate risk | Low risk | Low risk | Low risk | Low risk | Low risk | Low risk | Moderate risk |
| Yufei Zhang (2024) | Moderate risk | Low risk | Low risk | Low risk | Low risk | Low risk | Low risk | Moderate risk |
| Uğur Önal (2023) | Moderate risk | Low risk | Low risk | Low risk | Low risk | Low risk | Low risk | Moderate risk |
| Lumbreras-Iglesias (2024) | Moderate risk | Low risk | Low risk | Low risk | Low risk | Low risk | Low risk | Moderate risk |
| Markovska (2024) | Moderate risk | Low risk | Low risk | Low risk | Low risk | Low risk | Low risk | Moderate risk |
| Sotgiu (2018) | Moderate risk | Low risk | Low risk | Low risk | Low risk | Low risk | Low risk | Moderate risk |
| Liu (2021) | Moderate risk | Low risk | Low risk | Low risk | Low risk | Low risk | Low risk | Moderate risk |
| Jiao (2015) | Moderate risk | Low risk | Low risk | Low risk | Low risk | Low risk | Low risk | Moderate risk |
| Mammina (2013) | Low risk | Low risk | Low risk | Low risk | Low risk | Low risk | Low risk | Low risk |
| Cheng (2023) | Low risk | Low risk | Low risk | Low risk | Low risk | Low risk | Low risk | Low risk |
| Xiang (2024) | Moderate risk | Low risk | Low risk | Low risk | Low risk | Low risk | Low risk | Moderate risk |
| Gaspar (2022) | High risk | Low risk | Low risk | Low risk | Low risk | Low risk | Low risk | Moderate risk |
| Gregory (2010) | Moderate risk | Low risk | Low risk | Low risk | Low risk | Low risk | Low risk | Moderate risk |
| Li M (2025) | Low risk | Low risk | Low risk | Low risk | Low risk | Low risk | Low risk | Low risk |
| Xue Y (2025) | Moderate risk | Low risk | Low risk | Low risk | Low risk | Low risk | Low risk | Moderate risk |
| Zhao CF (2025) | High risk | Low risk | Low risk | Low risk | Low risk | Low risk | Low risk | Moderate risk |
| Onorato L (2022) | High risk | Low risk | Low risk | Low risk | Moderate risk | Low risk | Low risk | Moderate risk |
| Verma A (2022) | High risk | Low risk | Low risk | Low risk | Low risk | Low risk | Low risk | Moderate risk |
| Qiu M (2025) | Moderate risk | Low risk | Low risk | Low risk | Low risk | Low risk | Low risk | Moderate risk |
| Amir Mohammad Ali Tabrizi (2018) | High risk | Moderate risk | Low risk | Low risk | Low risk | Low risk | Low risk | Moderate risk |
| Melinte V (2025) | Moderate risk | Low risk | Low risk | Low risk | Moderate risk | Low risk | Low risk | Moderate risk |
| Cienfuegos-Gallet AV (2019) | Low risk | Low risk | Low risk | Low risk | Moderate risk | Low risk | Low risk | Moderate risk |
| Abu Jaber AMR (2024) | Moderate risk | Moderate risk | Low risk | Low risk | Low risk | Low risk | Low risk | Moderate risk |
| Machuca I (2019) | Low risk | Low risk | Low risk | Low risk | Moderate risk | Low risk | Low risk | Moderate risk |
| Yao Chen (2025) | Moderate risk | Low risk | Low risk | Low risk | Low risk | Low risk | Low risk | Moderate risk |
| Mouloudi E (2014) | Moderate risk | Low risk | Low risk | Low risk | Low risk | Low risk | Low risk | Moderate risk |
| Büyüktuna SA (2020) | Moderate risk | Low risk | Low risk | Low risk | Low risk | Low risk | Low risk | Moderate risk |
| Kong ZX (2022) | High risk | Moderate risk | Low risk | Low risk | Moderate risk | Low risk | Low risk | High risk |
| Wang J (2025) | Low risk | Low risk | Low risk | Low risk | Low risk | Low risk | Low risk | Low risk |
| Rojas LJ (2017) | Low risk | Low risk | Low risk | Low risk | Low risk | Low risk | Low risk | Low risk |
| Magobo RE (2023) | Low risk | Low risk | Low risk | Low risk | Moderate risk | Low risk | Low risk | Moderate risk |
| Capone A (2013) | Moderate risk | Low risk | Low risk | Low risk | Low risk | Low risk | Low risk | Moderate risk |
| Gomez-Simmonds (2015) | Moderate risk | Low risk | Low risk | Low risk | Low risk | Low risk | Low risk | Moderate risk |
| van Duin D (2015) | Moderate risk | Low risk | Low risk | Low risk | Low risk | Low risk | Low risk | Moderate risk |
