# Supplementary material for: Clinical Epidemiological Analysis of the Genotypic Spectrum and Mortality Risk in Carbapenem‐Resistant Klebsiella pneumoniae (CRKP) Infections
Source: Can J Infect Dis Med Microbiol. 2026 Jan 6;2026:1529426. doi: 10.1155/cjid/1529426 (PMC12771615; doi:10.1155/cjid/1529426)
Supplement: Supplementary file 4 — Supporting Information 4 Supporting Table 3: GRADE quality of evidence assessment for the main outcomes. [file CJID-2026-1529426-s004.docx]

| Supplementary Table 3 GRADE_Evidence_Quality | | | | | | | | |
| --- | --- | --- | --- | --- | --- | --- | --- | --- |
| Genotype/Group | Number of studies | Study design | Risk of bias | Inconsistency | Imprecision | Indirectness | Publication bias | Quality of evidence |
| KPC | 38 | Retrospective/Prospective cohort studies | Present | Moderate | Moderate | Low | Low | Moderate |
| OXA-48-like | 15 | Retrospective/Prospective cohort studies | Present | High | Moderate | Low | Low | Low |
| NDM/IMP/Other | 5 | Retrospective/Prospective cohort studies | Present | High | High | Low | Low | Low |
